# Design and Numerical Modeling of Terahertz Metasurface with Dual Functions of Sensing and Filtering

**DOI:** 10.3390/s24154823

**Published:** 2024-07-25

**Authors:** Lu Zhang, Huayan Sun, Zhe Chen, Runfeng Tang, Jinxiao Yang, Weilin Li

**Affiliations:** School of Information Science and Engineering, Yunnan University, Kunming 650000, China; lu_zhang0223@163.com (L.Z.); sunhuayan@stu.ynu.edu.cn (H.S.); tangrunfeng@stu.ynu.edu.cn (R.T.); yangjinxiao@stu.ynu.edu.cn (J.Y.); liweilin@stu.ynu.edu.cn (W.L.)

**Keywords:** terahertz, metasurface, multifunction, sensing element, filter

## Abstract

This study proposes a dual-functional terahertz device based on the Dirac semimetal, serving as both a sensing element and a band-pass filter. The device’s operating mode can switch between these two functions by utilizing the phase transition property of vanadium dioxide (VO_2_). When VO_2_ is in the insulating state, the device functions as a sensing element. The simulation results demonstrate an impressive refractive index sensitivity of 374.40 GHz/RIU (Refractive Index Unit). When VO_2_ is in the metallic state, the device functions as a band-pass filter, exhibiting a center frequency of 2.01 THz and a 3 dB fractional bandwidth of 0.91 THz. The integration of these dual functionalities within a single terahertz device enhances its utility in both sensing and filtering applications.

## 1. Introduction

Terahertz (THz) waves, with wavelengths ranging from 0.03 to 3 mm, bridge the gap between microwaves and infrared rays [1,2,3]. THz waves have unique properties that make them valuable in wireless communication, medical imaging, and security detection [4,5,6]. Metamaterials are composite artificial materials with distinct physical structures [7,8]. Metasurfaces, as two-dimensional metamaterials, can manipulate the amplitude, phase, and polarization of electromagnetic waves, enabling their widespread use in THz device designs [9,10,11].

THz sensors are important for environmental monitoring, medical detection, and other areas [12,13,14]. Materials like graphene, Dirac semimetals (DSMs), and vanadium dioxide (VO_2_) have advanced the development of metasurface sensors [15,16,17]. Among them, DSMs have a key characteristic of linear energy dispersion, leading to varied responses at different THz radiation frequencies. They exhibit a metallic response at lower frequencies, while their dielectric response dominates at higher frequencies [18]. In [19], Li et al. devised a three-band narrowband absorber based on a DSM, achieving a sensitivity of 152.5 GHz/RIU, which initially produced a highly sensitive sensor [19]. In [20], Wang et al. demonstrated a high-sensitivity sensor based on excellent absorption peaks, achieving a maximum sensitivity of 238 GHz/RIU [20]. In [21], Hou et al. introduced a BIC-based sensor based on a DSM with a sensitivity of 158 GHz/RIU [21]. Although integrating a DSM in metasurface devices can be a promising approach for THz sensor designs, it is still necessary to further improve sensor sensitivity. Moreover, fixed-function THz devices limit future applications of THz technology.

VO_2_, as a phase-change material, has been widely used in the design of multifunctional devices due to its ability to achieve a five-order-of-magnitude change in conductivity before and after the phase change [22]. Researchers have explored approaches by combining VO_2_ with a DSM to develop multifunctional THz devices. In [23], Wang et al. embedded VO_2_ into the dielectric layer and used its phase-change characteristics to achieve two functions of the asymmetric transmission and the bidirectional absorption [23]. In [24], Yi et al. proposed a multifunctional THz device based on a DSM and VO_2_ which has two absorption peaks when VO_2_ is in the metallic state and acts as a polarization converter when VO_2_ is in the insulating state [24]. In [25], Zhang et al. introduced a multifunctional absorber which switches from dual narrowband absorption to broadband absorption when VO_2_ changes from the insulating state to the metallic state [25]. These studies not only present multifunctional devices, but also further enrich the application of DSMs in THz devices.

As selective frequency devices in THz systems, THz filters play an essential role in filtering out unwanted frequency bands and noise [26,27,28]. However, there is limited research on filter design in existing multifunctional devices based on DSMs and VO_2_ [29], and it remains challenging to further improve sensing element sensitivity. 

This study presents a terahertz device that combines sensing and filtering functions using a DSM and VO_2_. When VO_2_ is in the insulating state, the device acts as a high-sensitivity sensing element based on the transmission dip and achieves a refractive index sensitivity of 374.40 GHz/RIU. When VO_2_ is in the metallic state, the device functions as a band-pass filter, with a center frequency of 2.01 THz and a 3 dB fractional bandwidth of 0.91 THz. This study not only improves the sensitivity of sensing element designed based on DSMs, but also extends the application of DSMs to filter designs. Furthermore, it introduces a new paradigm for multifunctional THz devices, enriching the design approach for THz devices.

## 2. Device Design

**Figure 1:**
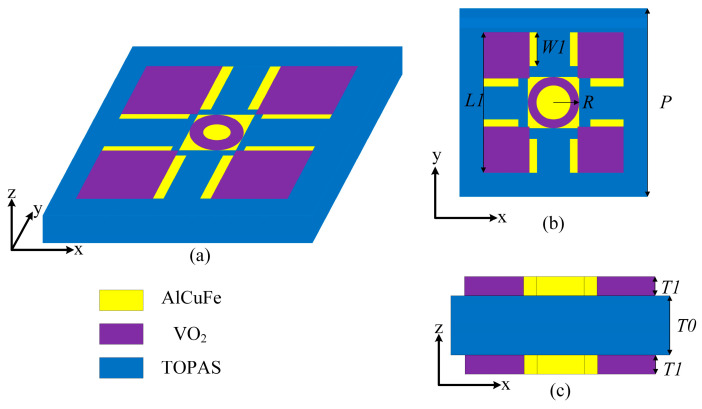
A unit schematic illustration of the terahertz sensing element/filter. (**a**) Front view of the device unit; (**b**) top view of the device; (**c**) side view of the device.

Figure 1 illustrates the unit structure of the switchable metasurface device utilizing VO_2_ and a DSM. The design employs a three-layer structure, as depicted in its front view in Figure 1c. Both the top and bottom layers share identical structures, incorporating a resonant design that integrates the DSM with VO_2_, as depicted in Figure 1b. The dielectric isolation layer is made of a TOPAS polymer with a dielectric constant ε_TOPAS_ = 2.35, which is a new type of optical thermoplastic material with properties such as high transmittance, low birefringence, and low absorption, making it an ideal choice for dielectric isolation layers in the design of terahertz optical components [30,31,32]. Several studies have shown that TOPAS can well support the fabrication of terahertz metasurfaces. For example, researchers have designed a dual-frequency polarization-tunable terahertz antenna based on graphene and TOPAS, demonstrating its potential in broadband terahertz devices [31]. In addition, efficient terahertz absorbers and other functional devices can be realized by the multilayer structure supported by a TOPAS substrate [33,34,35]. These studies have shown that TOPAS can not only support complex fabrication processes, but also provide the necessary mechanical strength while maintaining low losses [36,37]. In summary, TOPAS substrates can effectively support the fabrication process of our proposed ultra-thin devices. The thickness of each layer is specified as follows: *T*0 = 17.5 μm and *T*1 = 0.2 μm. Other geometric parameters of the device include the following: *P* = 90 μm, *W*1 = 20 μm, *L*1 = 80 μm, and *R* = 15 μm.

**Figure 2:**
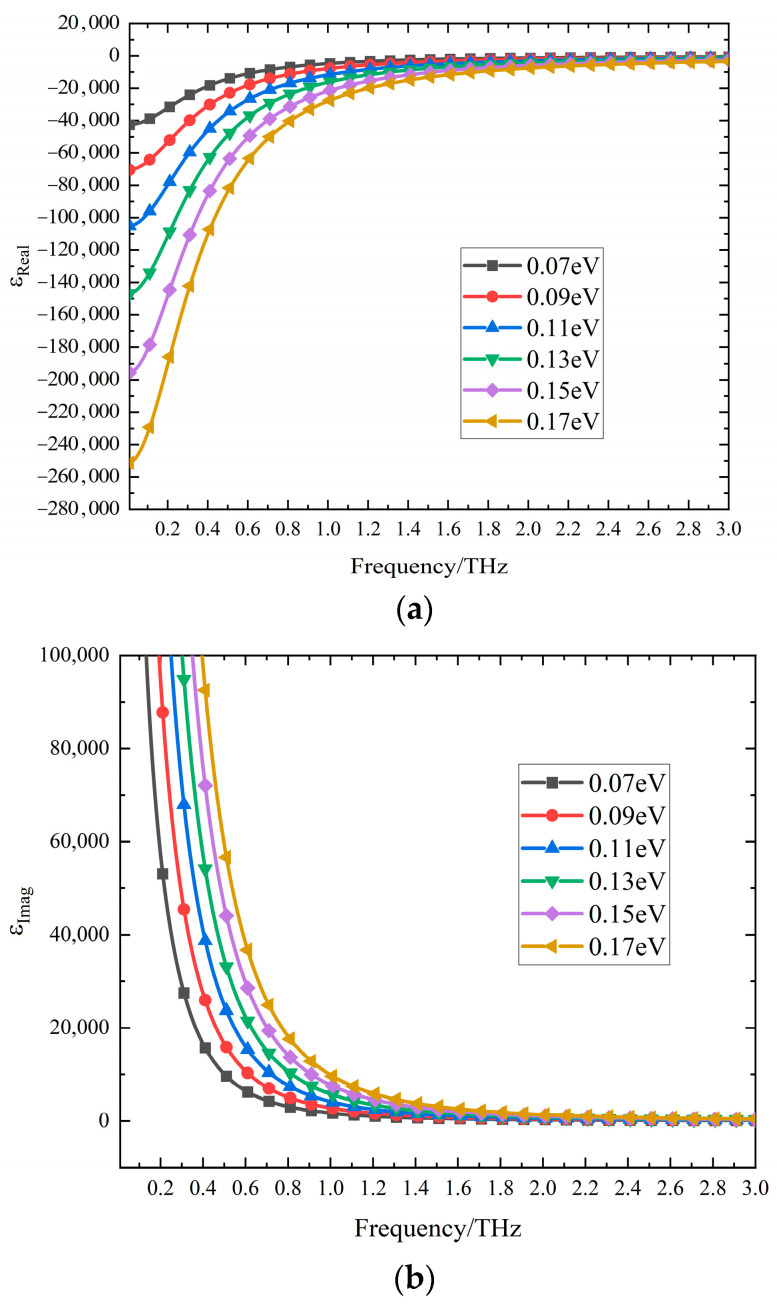
The variation in the relative dielectric constant of the DSM with Fermi energy level. (**a**) Real part; (**b**) imaginary part.

The complex conductivity of the DSM (AlCuFe) can be expressed using the Kubo formula under the long-wave limit condition, according to the random phase approximation theory. The real and imaginary parts of the conductivity can be calculated using the following equations [18]:

(1) Reσ{σ(Ω)}=e2ℏgkF24πΩGΩ/2 

(2)  Imσ{σ(Ω)}=e2ℏgkF24π24Ω1+π23TEF2+8Ω∫0εcGε − GΩ/2Ω2 − 4ε2εdε

where *e* is the charge quantity, $\;G\left(E\right)=\left(-E\right)\;-\;n(E)$, ${\varepsilon =E/E}_{F}$, $n\left(E\right)$ is the Fermi energy distribution function, for AlCuFe, the degeneracy factor is *g* = 40, $k_{F}{=E}_{F}/\hbar v_{F}\;$ is the Fermi momentum, *T* is the non-zero temperature, ***ℏ*** is the Planck constant, $v_{F}{=10}^{6}\;m/s$ is the Fermi velocity, $E_{F}$ is the Fermi energy level energy, Ω is the scattering rate, $\varepsilon_{C}{=E}_{C}/E_{F}$, where$\;E_{C}$ is the cutoff energy. Considering the interband electron transmission, the permittivity of the DSM can be expressed as [18]:

(3) $${\;\varepsilon =\varepsilon }_{b}+i\sigma /{\omega \varepsilon }_{0}$$

where $\varepsilon_{0}$ is the permittivity of the vacuum, $\varepsilon_{b}=1$ is the permittivity of the background, and σ is the conductivity of the DSM. As shown in Figure 2a,b, it can be clearly seen that both the Fermi level and frequency affect the permittivity of the DSM. Within the frequency range from 0 to 3 THz, the real part of the permittivity is negative and increases with frequency, while the imaginary part is positive and decreases with frequency. Simultaneously, as the Fermi level increases from 0.07 eV to 0.17 eV, at the same frequency, the real part of the DSM’s permittivity gradually decreases, while the imaginary part increases.

Furthermore, within the terahertz frequency range, the permittivity of VO_2_ can be effectively described by the Drude model, which considers the behavior of free carriers in the material. The Drude model provides a simplified yet accurate representation of the complex permittivity of materials with free charge carriers. The permittivity of VO_2_ can be expressed using the Drude model as follows [23]:

(4) $$\varepsilon \left(\omega \right){=\varepsilon }_{\infty }\;-\;\frac{\omega_{P}^{2}\left(\sigma_{{\mathrm{VO}}_{2}}\right)}{\omega^{2}+i\omega \gamma }\;$$

where $\varepsilon_{\infty }=12$ is the permittivity at infinite frequency, the collision frequency is $\gamma =5{.75\;\times \;10}^{13}$ rad/s, and the plasma frequency can be approximated as follows:

(5) $${\;\omega }_{P}^{2}\left(\sigma_{{\mathrm{VO}}_{2}}\right)=\frac{\sigma_{{\mathrm{VO}}_{2}}}{\sigma_{0}}\omega_{P}^{2}\left(\sigma_{0}\right)$$

where $\sigma_{0}{=3\;\times \;10}^{5}\;$ S/m and $\omega_{p}\left(\sigma_{0}\right)=1{.4\;\times \;10}^{15}\;$ rad/s. The conductivity *σ*₀ represents the DC conductivity of VO_2_ in the metallic state and reflects the ability of the material to conduct electricity at zero frequency. The plasma frequency $\omega_{p}\left(\sigma_{0}\right)$ describes the collective oscillatory properties of free electrons in the VO_2_ material [38,39]. During the simulation, we consider the conductivity of VO_2_ to be 10.0 S/m when it is in the insulating state and 200,000.0 S/m when it is in the metallic state. In practical applications, the temperature can be altered through heat to induce the transition of VO₂ from the insulating state to the metallic state, achieving the phase change of VO₂ [40].

In this investigation, we employed the electromagnetic simulation software CST Microwave Studio 2020, utilizing the frequency domain solver to accurately model and simulate the devised device. Terahertz waves were configured to propagate along the z-axis, perpendicular to the metasurface unit cell surface. Furthermore, periodic boundary conditions were applied in the x and y directions (unit cell) and the open (add space) boundary conditions in the z direction [41]. 

## 3. Results and Discussion

### 3.1. Sensing Element

**Figure 3:**
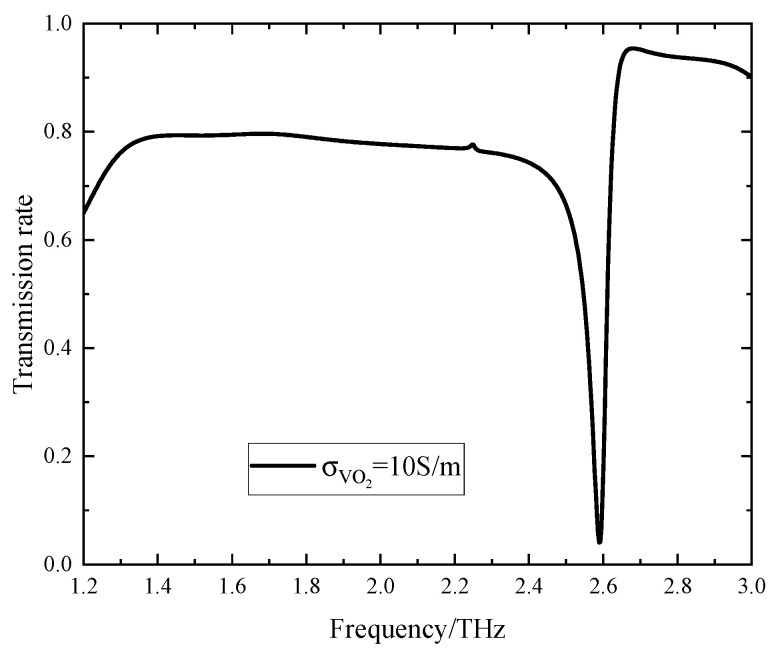
The transmission spectrum of the device when VO_2_ is in the insulating state.

When VO_2_ is in the insulating state and the Fermi energy level is 0.17 eV, the portion of the resonant layer comprising VO_2_ can be equated to gaps. Under these conditions, the transmission spectra of the device are illustrated in Figure 3. It is evident from the spectra that the device displays a significant transmission dip at 2.59 THz, characterized by a transmission spectrum as minimal as 0.04 and a high Q value of 69.97. Hence, the device can be effectively used as a terahertz sensing element when VO_2_ is in the insulating state.

**Figure 4:**
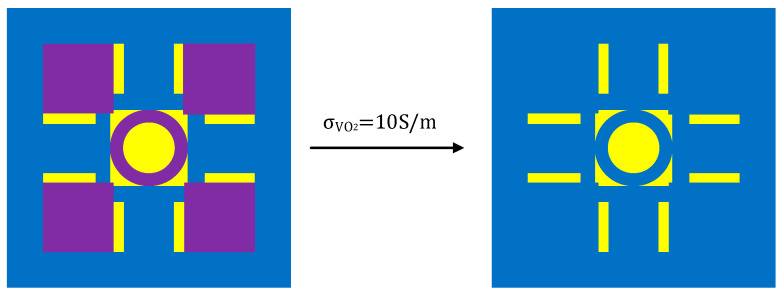
An equivalent model of the device when VO_2_ is in the insulating state.

When VO_2_ is in the insulating state, the circular ring in the central region of the device and the four square VO_2_ sections can be considered gaps. Therefore, the device can be interpreted as a resonant structure, as depicted in Figure 4. Thus, the device can be conceptually divided into two components: one component comprises a DSM with circular grooves situated at the center, while the other component consists of strip structures positioned around the periphery.

**Figure 5:**
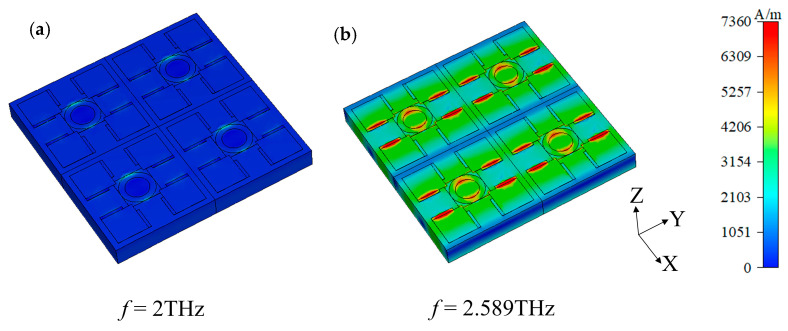
The magnetic field distribution of the sensing element at 2.00 THz and the transmission dip at 2.59 THz. (**a**) 2.00 THz; (**b**) 2.59 THz.

Magnetic field distributions can provide important information about electromagnetic resonance modes, and multiple resonance modes may exist in hypersurface structures. By observing the magnetic field distribution, these modes can be effectively distinguished and their nature understood, which is important for optimizing the design and improving sensor performance. In order to elucidate the physical mechanism that leads to a decrease in resonance when VO_2_ is in an insulating layer, the magnetic field distribution in the mode of the sensing element is analyzed in detail. The three-dimensional distribution of the magnetic field of the device is presented in Figure 5, when the device is in the sensing element mode. It is evident that at 2.00 THz, the magnetic field predominantly disperses along both sides of the central circle, exhibiting weak strength. Conversely, within the transmission dip at 2.59 THz, the magnetic field primarily concentrates within the inner region of the circular ring and on either side of the DSM strip structure along the y direction. This observation aligns with the equivalent structure depicted in Figure 4 of the device’s equivalent model under VO_2_’s insulating state. Hence, we can infer that the transmission dip of the device predominantly originates from the magnetic field excitation within the circular notch and the DSM strip structure.

**Figure 6:**
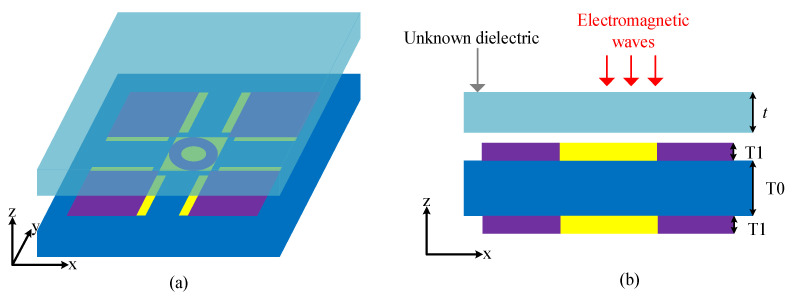
A schematic diagram of the sample covering the entire resonant structure (90 μm × 90 μm): (**a**) the front view; (**b**) the side view.

The resonant frequency of the device is sensitive to changes in the refractive index of external objects, leading to a shift in its resonant frequency corresponding to variations in the refractive index. Thus, we proceed to conduct an in-depth analysis of its sensing capabilities. Within the CST simulation software 2020, samples with varying refractive indices are positioned directly above the sensing element to emulate real-world scenarios of refractive index alterations during detection, as depicted in Figure 6a. The side view is shown in Figure 6b. Here, we set the sample thickness (t) to 15 μm, and the distance between the sample and the sensing element surface is 5 μm [42].

The following relationship exists between the permittivity ε and the refractive index n:

(6) $${\;\varepsilon =n}^{2}/\mu \;$$

Since most of the substances are non-magnetic, *μ* = 1. Therefore, objects with varying refractive indices are emulated in the simulation design by adjusting the permittivity of the samples under examination.

The main parameters to measure the performance of the sensing element are the quality factor *Q* and the sensitivity *S*, where *Q* is calculated as follows:

(7) $${Q=f}_{0}/\mathrm{FWHM}$$

where *f*_0_ is the center frequency of the resonant wave peak or dip, and *FWHM* is the half-peak full width of the wave dip or peak. When the sensing element’s sensitivity remains constant, a higher *Q* value indicates that changes in the resonant frequency are more easily detected, thereby leading to improved sensing performance.

The formula for *S* is as follows:

(8)  S=∆f/∆n

where *∆f* is the frequency shift of the resonant frequency with the change in the refractive index, and *∆n* is the change in the refractive index of the sample. The sensitivity (*S*) of the sensing element directly influences how responsive the resonant frequency is to changes in the refractive index. A higher sensitivity means that the sensing element can more effectively distinguish between objects with varying refractive indices, ultimately leading to improved sensing performance.

**Figure 7:**
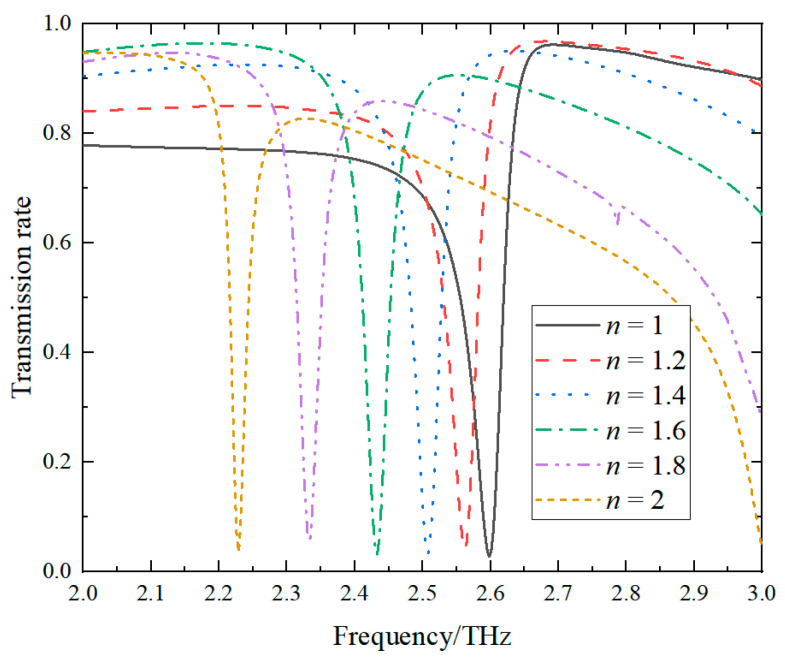
The transmission spectra of the sensing element as *n* increase from 1 to 2.

Various biomolecules have different refractive index ranges between 1.4 and 2 [43]. Hence, we set the refractive index of the sample between 1 and 2 for the refractive index sensitivity calculations in this study. The fixed sample thickness is 15 μm. Figure 7 illustrates the transmission spectra in the frequency range from 2 to 3 THz as the refractive index of the sample increases from 1 to 2. It is evident that the transmission dip shifts leftward with the increasing refractive index. This phenomenon arises from an alteration in the refractive index of the sample positioned on the sensing element’s surface, inducing a modification in the surrounding dielectric constant. Therefore, this metasurface can be used as a sensing element for monitoring resonant frequency shifts.

**Figure 8:**
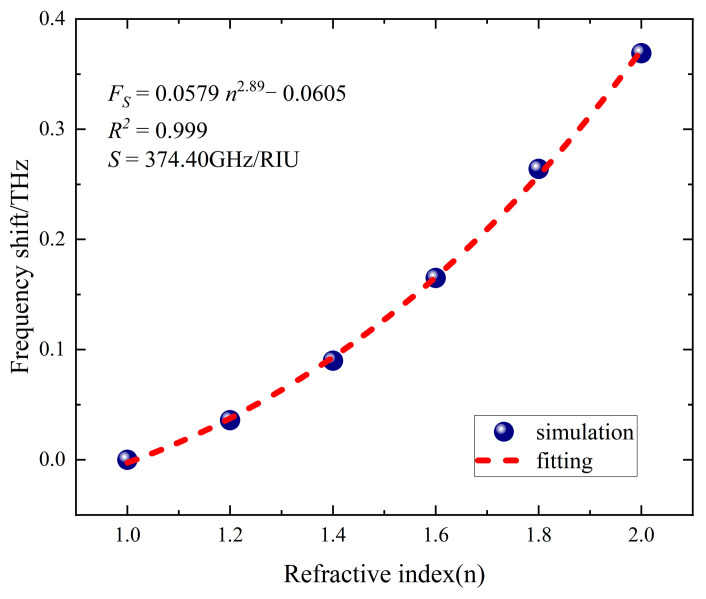
Frequency shift and fitting curves.

In order to elucidate the effect of the refractive index of the sample on the resonant frequency, Figure 8 shows the frequency shift of the resonance valley versus the refractive index of the sample *n*. By using an appropriate regression method, we effectively modeled the variation in resonant frequency according to the refractive index, where each circular data point in the figure represents the frequency offset and the dashed line represents the regression curve. It is clear from the figure that the resonance offset increases as the refractive index of the sample increases, showing a nonlinear relationship. We determined the frequency shift as $F_{s}=f-f_{0}$, where *f* denotes the resonant frequency when the metasurface covers the sample, and *f*_0_ denotes the resonant frequency when there is no sample coverage. The resonant frequency shift associated with the refractive index corresponds to a fit function of $F_{s}=0.06\;n^{2.89}-0.06$, with a fit coefficient *R*^2^ of 0.99. The sensitivity of the sensing element, *S*, can be defined as the relationship between the resonant frequency shift ($F_{s}$) and the change in refractive index (*Δn*), which results in a refractive index sensitivity *S* of the device of 374.40 GHz/RIU.

In addition, the sensitivity of the sensing element designed in this study was further compared with sensing elements based on DSMs in recent years, as shown in Table 1 [19,20,21,44,45], demonstrating the higher sensitivity of our sensing element.

**Figure 9:**
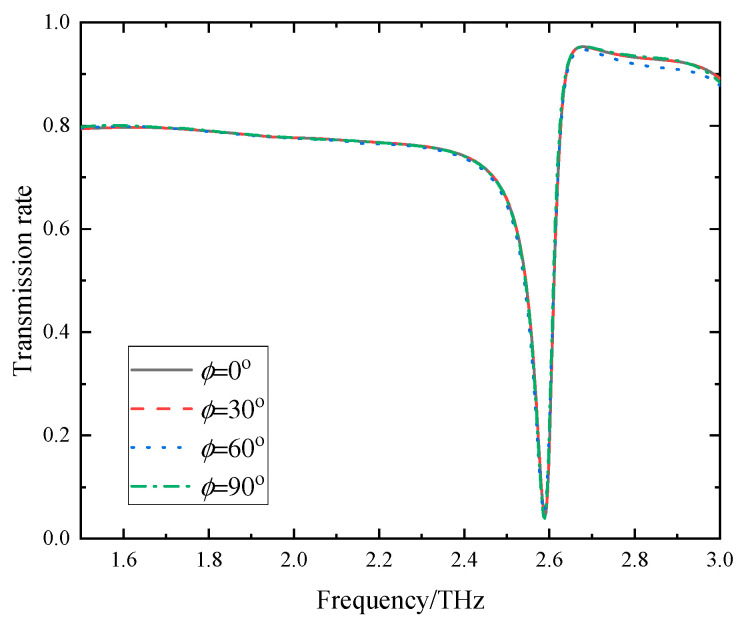
The transmission spectra of the sensing element with the polarization angle *ϕ.*

In practical sensing element applications, the influence of the incident electromagnetic wave’s polarization angle *ϕ* on performance is crucial. Therefore, the effect of the variation in the *ϕ* of the incident electromagnetic wave on the transmission spectra of the sensing element was investigated. Figure 9 illustrates the variation in the device’s transmission spectra as the polarization angle increases from 0° to 90°. Obviously, the transmission spectra remain unchanged despite variations in the polarization angle. Hence, the sensing element exhibits remarkable polarization-insensitive characteristics that can be attributed to the device’s structural symmetry.

### 3.2. Band-Pass Filter

**Figure 10:**
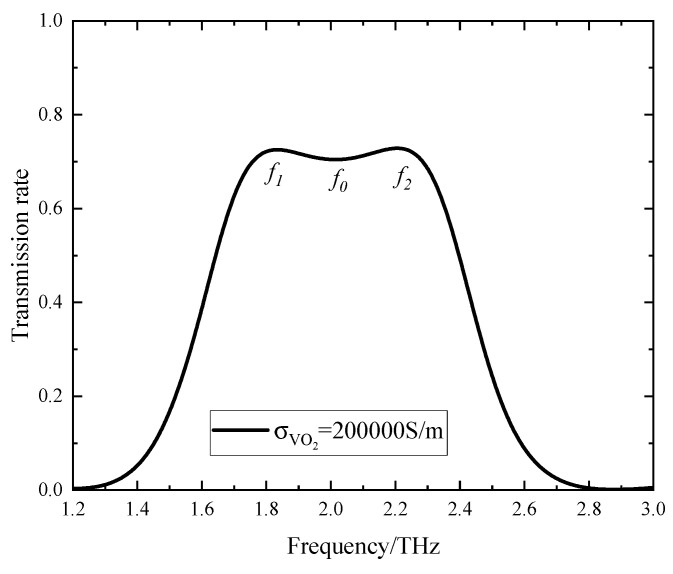
The transmission spectrum of the device when VO_2_ is in the metallic state.

When VO_2_ is in the metallic state and the Fermi energy level is 0.17 eV, the device functions as a band-pass filter, as depicted in Figure 10, which presents the transmission spectra. The band-pass filter exhibits a center frequency of 2.01 THz, a 3 dB fractional bandwidth of 0.91 THz, and a transmission spectrum of 0.70. Within the passband, two transmission peaks occur at frequencies *f*_1_ = 1.83 THz and *f*_2_ = 2.21 THz, both with a corresponding transmission spectrum of 0.73.

**Figure 11:**
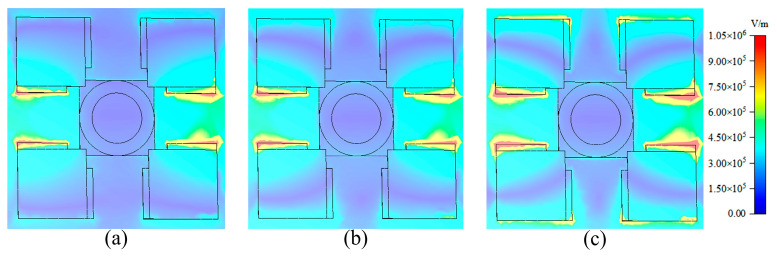
Electric field distribution in resonant layer. (**a**) *f*_1_; (**b**) *f*_0_; (**c**) *f*_2_.

Electric field distribution can provide detailed resonance mode information, identify critical regions, optimize resonance characteristics, improve filter performance, and provide strong support for designing equivalent circuits. To understand the physical mechanism of the proposed device functioning as a band-pass filter, the electric field distribution at the transmission peaks *f*_1_ and *f*_2_, along with the center frequency *f*_0_, was analyzed, as illustrated in Figure 11. The color bar displayed on the figure’s right side denotes the electric field intensity. Visibly, the predominant distribution of electric fields occurs within the gap of the resonant layer, where the DSM is employed. This suggests that the passband primarily arises due to the resonance within the resonant layer’s gap when the device operates as a band-pass filter.

**Figure 12:**
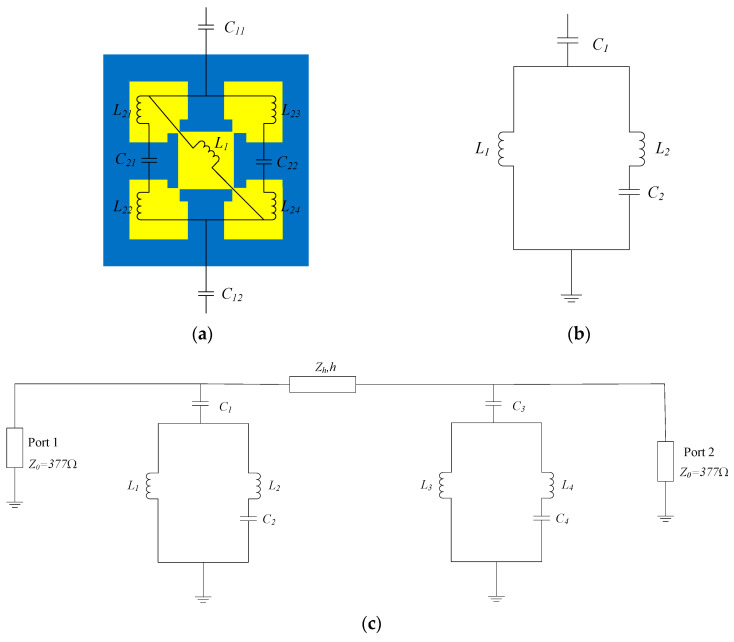
The equivalent circuit model of the band-pass filter. (**a**) The top and bottom equivalent circuit, Where the yellow part represents the VO_2_ structure in the metallic state; (**b**) the simplified circuit; (**c**) the complete equivalent circuit model.

The analysis of the electric field within the band-pass filter reveals that VO_2_ behaves similarly to metal in the metallic state. Thus, based on the electric field distribution, the equivalent circuit approach was adopted to further investigate the physical mechanism for broadband transmission when VO_2_ is in the metallic state. The unit equivalent circuit of the proposed device, illustrated in Figure 12a, characterizes the gaps between neighboring cells as capacitors *C*_11_ and *C*_12_, while the internal gaps within each cell are represented by capacitors *C*_21_ and *C*_22_. Additionally, the DSM regions correspond to inductors *L*_1_, *L*_21_, *L*_22_, *L*_23_, and *L*_24_. Due to the structural symmetry of the filter, it can be simplified into a circuit, as depicted in Figure 12b, where the relationship between the simplified equivalent circuit and the initial equivalent circuit adheres to the following conditions:

(9) $${\;L}_{2}=\frac{\left(L_{21}{+L}_{22}\right)\left(L_{23}{+L}_{24}\right)}{L_{21}{+L}_{22}{+L}_{23}{+L}_{24}}$$

(10) $$C_{1}=\frac{C_{11}C_{12}}{C_{11}{+C}_{12}}$$

(11) $$C_{2}{=C}_{21}{+C}_{22}$$

The input and output ports of this band-pass filter can be equated to port 1 and port 2, respectively, with a free-space wave impedance *Z*_0_ = 377 Ω. Since the top layer shares an identical resonance structure with the bottom layer, they are equated to the same circuit structure in the circuit model. Furthermore, the intermediate TOPAS dielectric layer is analogized to the transmission line with the characteristic impedance $Z_{h}=Z_{0}/\sqrt{\varepsilon_{r}}$. Hence, a comprehensive circuit model of the device has been developed, as depicted in Figure 12c. Subsequently, the transmission response of the equivalent circuit model undergoes simulation via the Advanced Design System 2020 (ADS 2020) software.

In a simulation of the equivalent circuit, the initial values of the components in the equivalent circuit can be obtained by the following equations [46]:

(12) $$\;\;\;\;\;L=\mu_{0}\frac{P}{2\pi }ln\left[\frac{1}{\sin\left(\frac{\pi b}{2P}\right)}\right]\;\;\;\;\;\;\;\;\;$$

(13) $$C=\varepsilon_{0}\varepsilon_{eff}\frac{2P}{\pi }ln\left[\frac{1}{\sin\left(\frac{\pi g}{2P}\right)}\right]\;\;\;$$

where *ε*_0_ is the permittivity of the free space, *ε_eff_* is the effective permittivity of the medium, *μ*_0_ is the permeability of the free space, *P* is the period length of the structure, *g* is the width of the gap, and *b* is the width of the resonant structure.

**Figure 13:**
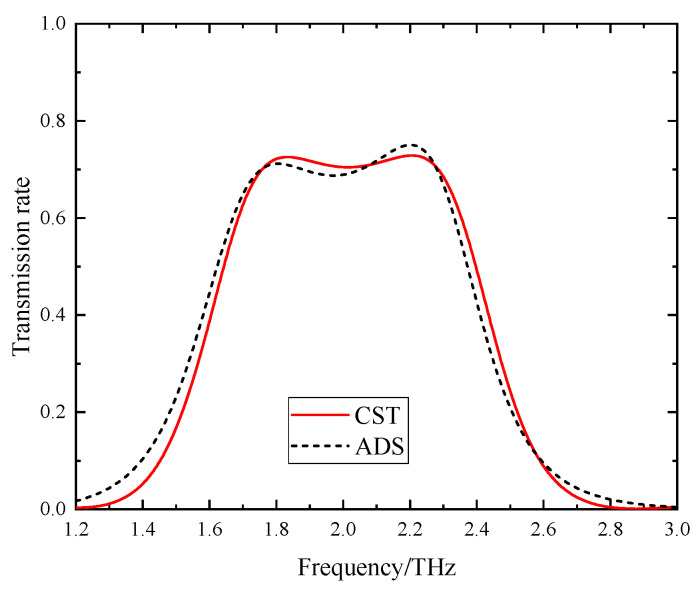
Transmission spectrum comparison.

The initial parameters for the simulation were configured within the equivalent circuit, and then, the parameters in the equivalent circuit were numerically optimized. The final fitted values for each component within the equivalent circuit are determined as follows: *Z_h_ =* 246.00 Ω, *C*_1_ *= C*_3_ *=* 2.46 fF, *C*_2_ *= C*_4_ *=* 0.33 fF, *L*_1_ *= L*_3_ *=* 13.30 pH, and *L*_2_ *= L*_4_ *=* 6.82 pH. The outcomes of the proposed band-pass filter configuration are presented in Figure 13, illustrating the simulation results generated by the ADS software. A comparative analysis between the equivalent circuit simulation and CST software simulation outcomes is depicted in the same figure. It is obvious that the equivalent circuit structure provides a precise match with the transmission response of the proposed band-pass filter. Thus, the transmission characteristics of the band-pass filter can be effectively replicated through the equivalent circuit model illustrated in Figure 12. Due to the structure’s symmetry when rotated by 90 degrees, a polarization-independent response is expected. Our simulations confirm this behavior.

### 3.3. The Impact of Parameters

**Figure 14:**
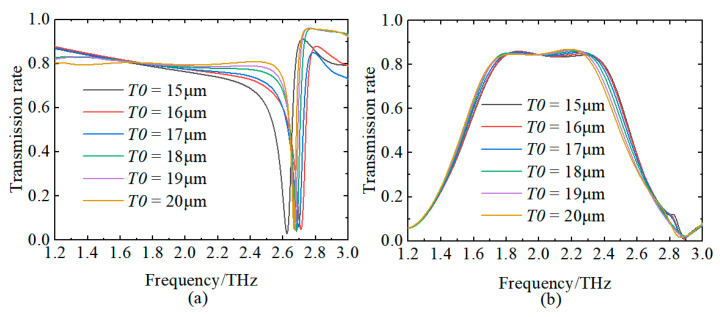
Effect of substrate thickness *T0* on transmission spectrum of device. (**a**) Sensing element; (**b**) band-pass filter.

In order to illustrate more clearly the effect of errors in the fabrication process and their uncertainties on the device, we use substrate thickness as an example. The substrate thickness *T*0 varies from 15 μm to 20 μm in 1 μm steps. Figure 14 illustrates the effect of this error on the transmission spectrum of the device. As can be seen in Figure 14a, the device shows significant transmission attenuation in the range of 2.57–2.62 THz, and the transmission spectra are all below 0.05. Therefore, even with some degree of error in substrate thickness, the device can still be used effectively as a terahertz sensing element. The error in substrate thickness has little effect on the performance of the sensing element but has a small effect on the resonant frequency. Figure 14b shows the effect of substrate thickness error on the band-pass filter. From the figure, it can be concluded that small uncertainties in the manufacturing process have almost no effect on the band-pass filter we designed.

## 4. Conclusions

We proposed a versatile, reconfigurable terahertz device based on a DSM and VO_2_. The device consists of a three-layer structure, wherein the top and bottom layers are made up of the same structure. The numerical simulation results show that the device is a high-sensitivity sensing element with a refractive index sensitivity of 374.40 GHz/RIU when VO_2_ is in the insulating state, and it is a band-pass filter with a center frequency of 2.01 THz and a 3 dB fractional bandwidth of 0.91 THz when VO_2_ is in the metallic state. The operating mechanism of the device was also analyzed through electromagnetic field analysis and equivalent circuit models. In addition, the device has excellent polarization-insensitive characteristics due to its symmetrical structure. This study not only expands the application of DSMs in filter design, but also integrates sensing element and filter functionalities, thus enriching the design of multifunctional terahertz devices. The research outcomes are promising for future applications in THz communication and imaging.

## Figures and Tables

**Table 1:** Comparison with previous sensing elements.

Reference	Sensitivity (GHz/RIU)
[19]	152.5
[20]	238
[21]	158
[44]	134.25
[45]	120
This work	374.40

## Data Availability

Data are contained within the article.
